# Effects of Acute Exposure to Increased Plasma Branched-Chain Amino Acid Concentrations on Insulin-Mediated Plasma Glucose Turnover in Healthy Young Subjects

**DOI:** 10.1371/journal.pone.0120049

**Published:** 2015-03-17

**Authors:** Sarah Everman, Lawrence J. Mandarino, Chad C. Carroll, Christos S. Katsanos

**Affiliations:** 1 Center for Metabolic and Vascular Biology, Arizona State University/Mayo Clinic in Arizona, Scottsdale, Arizona, United States of America; 2 School of Life Sciences, Arizona State University,Tempe, Arizona, United States of America; 3 Department of Physiology, Midwestern University, Glendale, Arizona, United States of America; INSERM/UMR 1048, FRANCE

## Abstract

**Background:**

Plasma branched-chain amino acids (BCAA) are inversely related to insulin sensitivity of glucose metabolism in humans. However, currently, it is not known whether there is a cause-and-effect relationship between increased plasma BCAA concentrations and decreased insulin sensitivity.

**Objective:**

To determine the effects of acute exposure to increased plasma BCAA concentrations on insulin-mediated plasma glucose turnover in humans.

**Methods:**

Ten healthy subjects were randomly assigned to an experiment where insulin was infused at 40 mU/m^2^/min (40U) during the second half of a 6-hour intravenous infusion of a BCAA mixture (i.e., BCAA; N = 5) to stimulate plasma glucose turnover or under the same conditions without BCAA infusion (Control; N = 5). In a separate experiment, seven healthy subjects were randomly assigned to receive insulin infusion at 80 mU/m^2^/min (80U) in association with the above BCAA infusion (N = 4) or under the same conditions without BCAA infusion (N = 3). Plasma glucose turnover was measured prior to and during insulin infusion.

**Results:**

Insulin infusion completely suppressed the endogenous glucose production (EGP) across all groups. The percent suppression of EGP was not different between Control and BCAA in either the 40U or 80U experiments (*P* > 0.05). Insulin infusion stimulated whole-body glucose disposal rate (GDR) across all groups. However, the increase (%) in GDR was not different [median (1st quartile – 3rd quartile)] between Control and BCAA in either the 40U ([199 (167–278) vs. 186 (94–308)] or 80 U ([491 (414–548) vs. 478 (409–857)] experiments (*P* > 0.05). Likewise, insulin stimulated the glucose metabolic clearance in all experiments (*P* < 0.05) with no differences between Control and BCAA in either of the experiments (*P* > 0.05).

**Conclusion:**

Short-term exposure of young healthy subjects to increased plasma BCAA concentrations does not alter the insulin sensitivity of glucose metabolism.

## Introduction

Increased concentrations of branched-chain amino acids (i.e., leucine, isoleucine, and valine) (BCAA) in the plasma of obese individuals was first described more than 40 years ago [1,2], and it has been a common observation in obesity since then [3–6]. An apparent inverse association has now emerged between plasma BCAA and insulin sensitivity [7]. The plasma concentrations of specific BCAA, such as valine, show significant positive correlation with the homeostatic model assessment (HOMA) of insulin resistance [8], and an overall increase in the BCAA has been described as contributor to the insulin-resistant state that accompanies human obesity [4]. Furthermore, the improvement in insulin sensitivity after gastric bypass in obese individuals is observed together with a decrease in the plasma BCAA concentrations [9]. Such lines of evidence point to a possible causal link between increased plasma BCAA concentrations and insulin resistance. However, all this evidence is only descriptive in nature and cannot establish a cause-and-effect relationship between increased blood BCAA concentrations and decreased insulin sensitivity in humans.

Acute infusion of an amino acid mixture that includes the BCAA and results in approximately 2-fold increase in the plasma BCAA concentrations, impairs the insulin-stimulated whole-body glucose disposal in young healthy subjects [10,11]. Studies in rodents show that, although supplementation of a high-fat diet with BCAA prevents weight gain, these rats still develop insulin resistance in a manner similar to weight-gaining rats on just the high-fat diet, suggestive of an independent role of BCAA in inducing insulin resistance [4]. Overall, these lines of evidence indicate that increased plasma BCAA concentrations may have adverse effects on the regulation of plasma glucose homeostasis.

Our current knowledge linking the plasma BCAA to insulin resistance in humans is based on simple association studies. In addition to a possible role of BCAA in modifying glucose homeostasis, BCAA have a well-described positive role in maintaining muscle protein turnover [12,13]. Therefore, any information about a causal role of BCAA in altering insulin sensitivity of glucose metabolism in humans is of both physiological and clinical importance. An experimental approach where the insulin sensitivity is evaluated in the presence of acute exposure to increased plasma BCAA concentrations can directly address the short-term effects of increased plasma BCAA concentrations on hindering insulin sensitivity.

## Materials and Methods

### Study participants

All study procedures were approved by the Institutional Review Board at Arizona State University. Subjects were screened over the phone and those with body mass index > 30 kg/m^2^, diabetes, high blood pressure, heart disease, peripheral vascular disease, history of liver or kidney disease, smoking and use of either prescription or over-the-counter medications or ingestion of any supplements were excluded from participation in the study. Subjects included in the study were brought to the Clinical Research Unit (CRU) at Arizona State University for further screening. Those determined to be healthy based on standard medical history questionnaire, physical examination, resting electrocardiogram, and blood and urine tests, were randomly assigned into the following experiments designed to evaluate whole-body glucose turnover: 1) infusion of insulin at 40 mU/m^2^/min (40U) and in association with intravenous infusion of either branched-chain amino acids (BCAA group, N = 5) or saline (Control group; N = 5), 2) infusion of insulin at 80 mU/m^2^/min (80U) and in association with intravenous infusion of either BCAA (BCAA group; N = 4) or saline (Control group; N = 3). Morphological and biochemical parameters of the subjects studied in the BCAA and Control groups are shown in Table 1. The purpose and the design of the studies, as well as known risks associated with the study procedures, were explained to each subject and written informed consent was obtained.

**Table 1:** Physical and clinical characteristics of the subjects.

	Basal BCAA (2F/6M)	High BCAA (5F/4M)
Age, y	22.9 ± 1.7	19.1 ± 0.8
Weight, kg	68.8 ± 2.6	64.5 ± 3.4
Height, m	1.73 ± 0.03	1.70 ± 0.03
BMI, kg/m^2^	22.9 ± 0.6	22.4 ± 1.0
Body fat, %	13.2 ± 3.0	16.7 ± 1.7
Plasma cholesterol, mg/dl	136.6 ± 5.6	155.1 ± 11.1
Plasma HDL-C, mg/dl	48.7 ± 4.5	56.3 ± 4.7
Plasma triglycerides, mg/dl	67.7 ± 6.7	70.9 ± 9.2
ALT, IU/l	19.6 ± 2.7	23.2 ± 2.9
AST, IU/l	24.6 ± 2.4	26.0 ± 2.9
Plasma glucose, mg/dl	84.0 ± 2.3	85.2 ± 2.5
Plasma insulin, uIU/ml	4.5 ± 0.6	3.9 ± 0.8
HOMA-IR	0.9 ± 0.1	0.8 ± 0.2
Hemoglobin A1C (%)	5.1 ± 0.1	5.1 ± 0.1

Values are means ± SE; BMI, body mass index, ALT, alanine aminotransferase; AST, aspartate aminotransferase; BCAA, branched-chain amino acids; HDL-C, High Density Lipoprotein-Cholesterol; HOMA-IR, Homeostatic model assessment of insulin resistance; Body fat was determined using bioimpedance; There were no significant differences between groups (*P* >0.05).

### Experimental protocol

**Fig 1:**
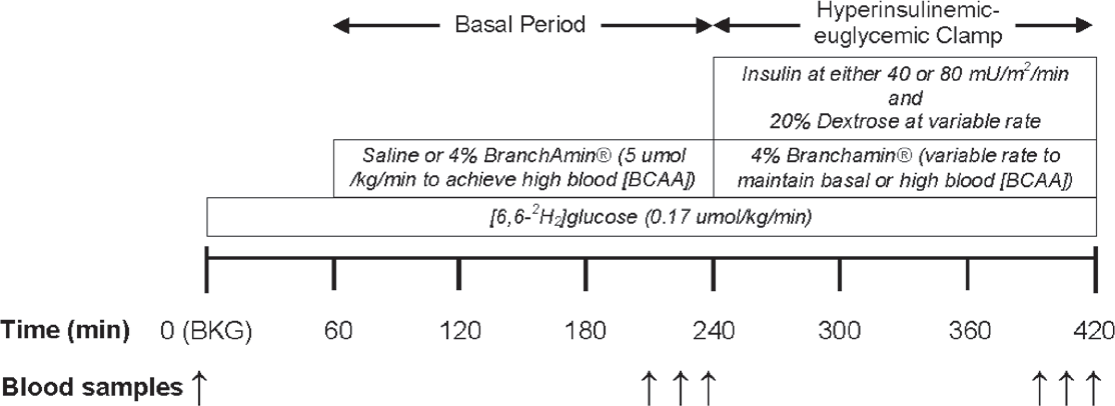
Experimental design. Infusion protocol depicting the “basal” and “hyperinsulinemic-euglycemic clamp” study periods described in the text. [6,6–^2^H_2_]glucose, branched-chain amino acids (BCAA; i.e., BranchAmin), saline, insulin and glucose were infused as indicated; [BCAA], total branched-chain amino acids concentration.

Prior to participation in the infusion experiment, subjects were instructed to abstain from alcohol consumption, any form of exercise, and keep their normal diet for the three-day period before the experiment. Subjects were asked to fast starting at 10 PM the night before the infusion experiment and arrive at the CRU at approximately 7:00 AM the following morning, at which time compliance with the above instructions was verbally verified. A catheter was inserted into an antecubital vein of their arm for infusions, while a second catheter was inserted in a hand vein for blood sampling using the heated hand technique. An infusion of [*6*,*6–* ^*2*^ *H*_*2*_]glucose (prime: 16.7 umol/kg; rate: 0.17 umol/kg/min), was initiated after the collection of blood samples for the determination of background stable isotope enrichment and biochemical parameters. The experimental protocol is depicted in Fig. 1. Subjects in the BCAA group received BCAA (4% BranchAmin; Baxter, Deerfield, IL; 1.38 g isoleucine, 1.38 g leucine, and 1.24 g valine per 100 ml) infusion at a rate of 5 umol/kg/min (prime: 15 umol/kg/min over 30 mins) to increase the concentration of plasma BCAA. After three hours of either BCAA or saline infusion (i.e., Control), insulin infusion was started at a rate of either 40 mU/m^2^/min (prime: 80 mU/m^2^/min over 10 mins) or 80 mU/m^2^/min (prime: 160 mU/m^2^/min over 10 mins) to stimulate whole-body glucose turnover. Variable rate of 20% dextrose was infused simultaneously with the insulin to maintain the plasma glucose concentration at that measured at the end of the three-hour basal period (i.e., hyperinsulinemic-euglycemic clamp). The dextrose solution was enriched with 1.8% [*6*,*6–* ^*2*^ *H*_*2*_]glucose to minimize changes in plasma glucose isotopic enrichment during the hyperinsulinemic-euglycemic clamp. Because insulin infusion decreases the plasma amino acid concentrations, we infused 4% BranchAmin at a variable rate during the hyperinsulinemic-euglycemic clamp in all infusion experiments to maintain the plasma BCAA at the levels observed during the basal period (i.e., the end of the three hours of BCAA or saline infusions). A drop in amino acid levels below those observed in basal/postabsorptive conditions creates a metabolic circumstance associated with increased plasma glucose turnover [14].

Blood samples were collected prior to the initiation of the infusions, during the last 30 mins of the basal period, associated with either the BCAA or saline infusions, as well as during the last 30 mins of the hyperinsulinemic-euglycemic clamp. Blood samples were collected for determinations of plasma glucose concentration and enrichment, as well as insulin and amino acid concentrations. Plasma glucose concentrations were determined every 5–10 mins during the hyperinsulinemic-euglycemic clamp in order to adjust the rate of 20% dextrose infusion to maintain the plasma glucose at the levels measured prior to the initiation of the insulin infusion. BCAA concentrations were also determined every 20–30 mins during the hyperinsulinemic-euglycemic clamp with the purpose to adjust the rate of 4% BranchAmin to maintain the plasma total BCAA levels as close to those measured prior to the initiation of the insulin infusion.

### Analyses of samples

Plasma glucose and BCAA concentrations during the hyperinsulinemic-euglycemic clamp were measured using an automated glucose analyzer (YSI 2300, Yellow Springs, OH), and a rapid spectrophotometric assay [15], respectively. Plasma insulin concentrations were determined using a commercially available kit (ALPCO Diagnostics, Windham, NH). Plasma amino acid concentrations were determined using high-performance-liquid-chromatography (HPLC). With respect to the determination of the plasma amino acid concentrations, proteins in the plasma samples were precipitated with an equal volume of 10% trichloroacetic acid and the samples were derivatized with o-phthalaldehyde. Amino acids were separated using HPLC (LC-20AT and SIL-20ACHT; Shimadzu Scientific Instruments), and analyzed using fluorometric detection [16].

Plasma [*6*,*6–* ^*2*^ *H*_*2*_]glucose enrichment was measured as previously described [17]. Briefly, after the pentaacetate derivative of glucose was prepared, samples were analyzed using electron impact gas chromatography-mass spectrometry (Tracer GC Ultra-DSQ GG-MS; Thermo Scientific). Selected ion monitoring was performed for ions at mass-to-charge ratio (*m/z*) 200, 201, and 202, and correction was made for the contribution of singly labeled *m/z* 201 to the measured *m/z* 202. Enrichment was expressed as tracer-to-tracee ratio (TTR).

### Calculations

Steady-state equations were used to calculate rates of whole-body appearance (Ra) and disappearance (Rd) of glucose in the two periods representing the basal (i.e., no insulin) and insulin-stimulated conditions, and by averaging the enrichments measured at the end of each study period. The glucose kinetics were also calculated by using the non-steady-state Steel equation [18] modified for the use with stable isotope tracers [19]. However, these findings were not different from those when the glucose Ra and Rd were calculated using the steady-state equations, and therefore only data using steady-state equations are reported.

Whole-body glucose Ra was calculated as the ratio of the [*6*,*6–* ^*2*^ *H*_*2*_]glucose infusion rate (F) to the measured TTR (i.e., Ra = F/TTR). During the hyperinsulinemic-euglycemic clamp the rate of [*6*,*6–* ^*2*^ *H*_*2*_]glucose infused as part of the infusion of the 20% dextrose solution was included in the F for the calculations of whole-body Ra and Rd. Under steady-state conditions the whole-body glucose Rd equals that of Ra. Whole-body Rd describes the whole-body glucose disposal rate (GDR). Endogenous glucose production (EGP) was calculated in the insulin-stimulated condition by subtracting the exogenous glucose infusion rate from the whole-body glucose Ra. The glucose metabolic clearance rate was calculated as the ratio of whole-body glucose Rd to the plasma glucose concentration. Whole-body GDR was also adjusted to the prevailing steady-state plasma insulin concentrations [20], to obtain a more accurate measure of the insulin-mediated whole-body GDR.

Whole-body sensitivity to the insulin-mediated glucose disposal was calculated as the percent change of the GDR relative to that at basal: (GDR_insulin-stimulated_-GDR_basal_)/GDR_basal_ x 100. The sensitivity of insulin in suppressing the endogenous glucose production (i.e., hepatic insulin sensitivity) was calculated as: (EGP_basal_-_EGPinsulin-stimulated_)/EGP_basal_ x 100. In the basal period only, hepatic insulin sensitivity was also calculated across all nine subjects exposed to the increased plasma BCAA concentrations and all eight subjects serving as Controls as the product of EGP and plasma insulin concentration [21].

### Statistical analyses

Statistical analyses were performed using non-parametric tests because these tests do not make any assumptions regarding the distribution of the data. The Wilcoxon signed-rank test (i.e., two dependent samples) and the Friedman test (i.e., three dependent samples) were used to test for differences across study periods. Differences between groups at a given study period were analyzed with the Mann-Whitney test. Data are provided as mean ± SEM, as well as median (1^st^ quartile—3^rd^ quartile). Differences were considered statistically significant at *P* ≤ 0.05. Statistical analyses were performed using the Minitab 16 statistical analysis software (Minitab Inc., State College, PA).

## Results

### Plasma concentrations of amino acids, glucose and insulin

There were no significant differences (*P* >0.05) in the fasting concentrations (umol/l) of various amino acids between the BCAA and the Control subjects (arginine, 75±11, 74 (43–106) vs 65±8, 60 (46–87); asparagine, 59±4, 61 (48–69) vs 55±8, 54 (33–78); aspartate, 39±6, 35 (28–40) vs 31±3, 32 (24–38); glutamate, 88±7, 83 (79–103) vs 73±9, 69 (47–101); isoleucine, 65±9, 59 (43–85) vs 63±8, 58 (48–75); leucine, 101±20, 89 (57–150) vs 88±14, 81 (54–109); methionine, 48±6, 53 (29–58) vs 44±3, 43 (37–46); phenylalanine, 67±6, 61 (54–83) vs 62±6, 58 (47–79); serine, 99±12, 91 (74–114) vs 98±18, 93 (49–147); tyrosine, 84±7, 77 (68–98) vs 100±5, 106 (83–111); valine, 184±28, 168 (124–240) vs 164±22, 165 (111–223)). As expected due to the experimental design, the concentration of plasma BCAA increased relative to their fasting value only in the BCAA groups, and both in the basal period and during the hyperinsulinemic-euglycemic clamp. Plasma BCAA concentrations prior to the initiation of the BCAA or saline infusions (i.e., Time 0) as well as in the basal period and the period associated with the hyperinsulinemic-euglycemic clamp are shown for the 40U and 80U experiments in Tables 2 and 3, respectively. As also expected, the plasma BCAA concentrations were different between the BCAA and Control groups in the basal period and during the hyperinsulinemic-euglycemic clamp (*P* <0.05). The rate of BCAA infusion (umol/kg/min) to maintain the plasma BCAA concentrations during the hyperinsulinemic-euglycemic clamp at levels measured prior to the initiation of the insulin infusion (i.e., basal period) were 1.9 ± 0.6, 1.2 (1.2–3.0) and 6.0 ± 0.5, 5.5 (5.0–7.3) in the Control and BCAA groups, respectively, in the 40U experiment, and 3.7 ± 1.1, 4.6 (1.5–4.9) and 11.3 ± 2.3, 10.2 (7.6–16.0) in the Control and BCAA groups, respectively, in the 80U experiment.

**Table 2:** Plasma branched-chain amino acid concentrations in the study associated with the infusion of insulin at 40 mU/m^2^/min.

	Study Period		
	Time 0	Basal Period	Hyperinsulinemic-euglycemic Clamp
Isoleucine (umol/l)			
Control	62 ± 12 57 (43–84)	53 ± 11 42 (39–72)	56 ± 7 47 (43–74)
BCAA	77 ± 10 73 (59–98)	292 ± 47* † 252 (238–366)	221 ± 37* † 192 (151–305)
Leucine (umol/l)			
Control	86 ± 23 73 (48–132)	75 ± 19 62 (43–113)	59 ± 11^#^ 48 (40–83)
BCAA	114 ± 22 95 (85–151)	268 ± 60* † 212 (204–361)	195 ± 36* † 172 (133–268)
Valine (umol/l)			
Control	144 ± 30 130 (91–203)	129 ± 30 93 (86–189)	123 ± 18 122 (90–156)
BCAA	184 ± 29 168 (136–239)	516 ± 84* † 438 (414–657)	479 ± 63* † 406 (373–621)
BCAA (umol/l)			
Control	97 ± 21 94 (61–135)	85 ± 20 71 (56–122)	79 ± 12 72 (58–104)
BCAA	125 ± 20 112 (93–163)	359 ± 63* † 298 (287–461)	298 ± 45* † 257 (219–398)

Values are means ± SE and median (1^st^ quartile—3^rd^ quartile); Time 0, prior to the initiation of any infusions; Basal Period, infusion of saline (Control, N = 5) or branched-chain amino acids (BCAA, N = 5); Hyperinsulinemic-euglycemic Clamp, infusion of 40 mU/m^2^/min insulin together with variable rate of 20% dextrose to maintain the plasma glucose concentrations at those measured at the end of the Basal Period.

*Statistically different compared to Time 0 (*P* ≤0.05)

^#^Statistically different compared to Time 0 and Basal Period (*P* ≤0.05)

†Statistically different between Control and BCAA groups (*P* ≤0.05)

**Table 3:** Plasma branched-chain amino acid concentrations in the study associated with the infusion of insulin at 80 mU/m^2^/min.

	Study Period		
	Time 0	Basal Period	Hyperinsulinemic-euglycemic Clamp
Isoleucine (umol/l)			
Control	65 ± 8 61 (53–80)	55 ± 11 49 (40–75)	94 ± 20 97 (59–127)
BCAA	51 ± 15 43 (27–81)	254 ± 27* † 253 (202–305)	342 ± 96* † 263 (220–544)
Leucine (umol/l)			
Control	91 ± 13 89 (70–114)	61 ± 7 58 (49–74)	84 ± 11 75 (72–106)
BCAA	86 ± 38 57 (34–168)	231 ± 43* † 207 (167–318)	305 ± 79* † 248 (199–469)
Valine (umol/l)			
Control	198 ± 21 177 (177–239)	151 ± 8 151 (138–165)	207 ± 32 199 (155–266)
BCAA	184 ± 58 140 (104–307)	464 ± 33* † 463 (401–528)	670 ± 143* † 542 (504–963)
BCAA (umol/l)			
Control	118 ± 13 109 (100–144)	89 ± 7 90 (76–100)	128 ± 21 124 (95–166)
BCAA	107 ± 37 80 (56–185)	316 ± 33* † 308 (259–381)	439 ± 106* † 347 (312–659)

Values are means ± SE and median (1^st^ quartile—3^rd^ quartile); Time 0, prior to the initiation of any infusions; Basal Period, infusion of saline (Control, N = 3) or branched-chain amino acids (BCAA, N = 4); Hyperinsulinemic-euglycemic Clamp, infusion of 80 mU/m^2^/min insulin together with variable rate of 20% dextrose to maintain the plasma glucose concentrations at those measured at the end of the Basal Period.

*Statistically different compared to Time 0 (*P* ≤0.05)

†Statistically different between Control and BCAA groups (*P* ≤0.05).

Plasma glucose concentrations in the 40U and 80U experiments are depicted in Tables 4 and 5, respectively. In the 40U experiment, although no statistically significant differences were found in plasma glucose concentration across the three study periods in Control, there was a trend for significant difference in the BCAA group (*P* <0.07), which was associated with a trend for lower plasma glucose concentration in the basal period when compared to Time 0 (*P* <0.06). There were no differences in the plasma glucose concentration between groups at any of the study periods (*P* >0.05) in the 40U experiment. There were no differences in plasma glucose concentrations in either the Control or the BCAA group overtime in the 80U experiment, and no differences were detected either between groups at any of the study periods (*P* >0.05).

**Table 4:** Plasma glucose and insulin concentrations in the study associated with the infusion of insulin at 40 mU/m^2^/min.

	Study Period		
	Time 0	Basal Period	Hyperinsulinemic-euglycemic Clamp
Glucose (mg/dl)			
Control	87.4 ± 2.7 89.1 (81.5–92.2)	88.2 ± 1.9 87.4 (84.7–92.1)	91.3 ± 8.0 87.5 (78.9–105.5)
BCAA	90.4 ± 1.7 90.7 (86.7–94.0)	84.1 ± 2.4 85.3 (79.4–88.3)	85.0 ± 3.1 82.3 (79.4–92.1)
Insulin (uIU/ml)			
Control	5.2 ± 0.6 4.9 (4.1–6.6)	6.0 ± 1.8 4.6 (2.4–10.2)	50.3 ± 4.0* 48.5 (42.5–58.9)
BCAA	4.6 ± 1.2 3.6 (2.8–6.8)	6.3 ± 1.3 5.8 (3.8–9.0)	51.2 ± 2.5* 48.3 (46.7–57.2)

Values are means ± SE and median (1^st^ quartile—3^rd^ quartile); Time 0, prior to the initiation of any infusions; Basal Period, infusion of saline (Control, N = 5) or branched-chain amino acids (BCAA, N = 5); Hyperinsulinemic-euglycemic Clamp, infusion of 40 mU/m^2^/min insulin together with variable rate of 20% dextrose to maintain the plasma glucose concentrations at those measured at the end of the Basal Period;

*P ≤0.05 when compared to Time 0 and Basal Period.

**Table 5:** Plasma glucose and insulin concentrations in the study associated with the infusion of insulin at 80 mU/m^2^/min.

	Study Period		
	Time 0	Basal Period	Hyperinsulinemic-euglycemic Clamp
Glucose (mg/dl)			
Control	78.3 ± 0.5 78.3 (77.5–79.1)	81.0 ± 0.8 81.7 (79.5–81.9)	92.5 ± 7.8 85.3 (84.1–108.0)
BCAA	78.8 ± 2.5 77.3 (75.1–84.0)	72.5 ± 2.8 71.4 (67.9–78.4)	80.3 ± 4.9 80.7 (71.2–89.1)
Insulin (uIU/ml)			
Control	3.1 ± 0.8 2.9 (1.9–4.6)	5.0 ± 2.1 5.4 (1.3–8.5)	91.9 ± 10.8^&^ 101.1 (70.4–104.1)
BCAA	3.0 ± 0.7 2.8 (1.9–4.4)	4.6 ± 0.9 4.8 (2.8–6.3)	117.2 ± 7.0* 119.9 (103.2–128.6)

Values are means ± SE and median (1^st^ quartile—3^rd^ quartile); Time 0, prior to the initiation of any infusions; Basal Period, infusion of saline (Control, N = 3) or branched-chain amino acids (BCAA, N = 4); Hyperinsulinemic-euglycemic Clamp, infusion of 80 mU/m^2^/min insulin together with variable rate of 20% dextrose to maintain the plasma glucose concentrations at those measured at the end of the Basal Period;

*P ≤0.05 when compared to Time 0 and Basal Period

^&^ *P* = 0.09 when compared to Time 0 and Basal Period

As expected based on the experimental design, plasma insulin concentrations increased in both the Control and BCAA groups, and in both the 40U and 80U experiments during the hyperinsulinemic-euglycemic clamp. In the 40U experiment, insulin infusion during the hyperinsulinemic-euglycemic clamp significantly increased the plasma insulin concentrations in both the Control and BCAA group compared to basal period and Time 0 (Table 4). Plasma insulin concentrations increased also in the 80U experiment during the hyperinsulinemic-euglycemic clamp relative to the concentrations in the two other study periods, and in both the Control and BCAA group (Table 5).

### Whole-body glucose disposal and endogenous glucose production

**Fig 2:**
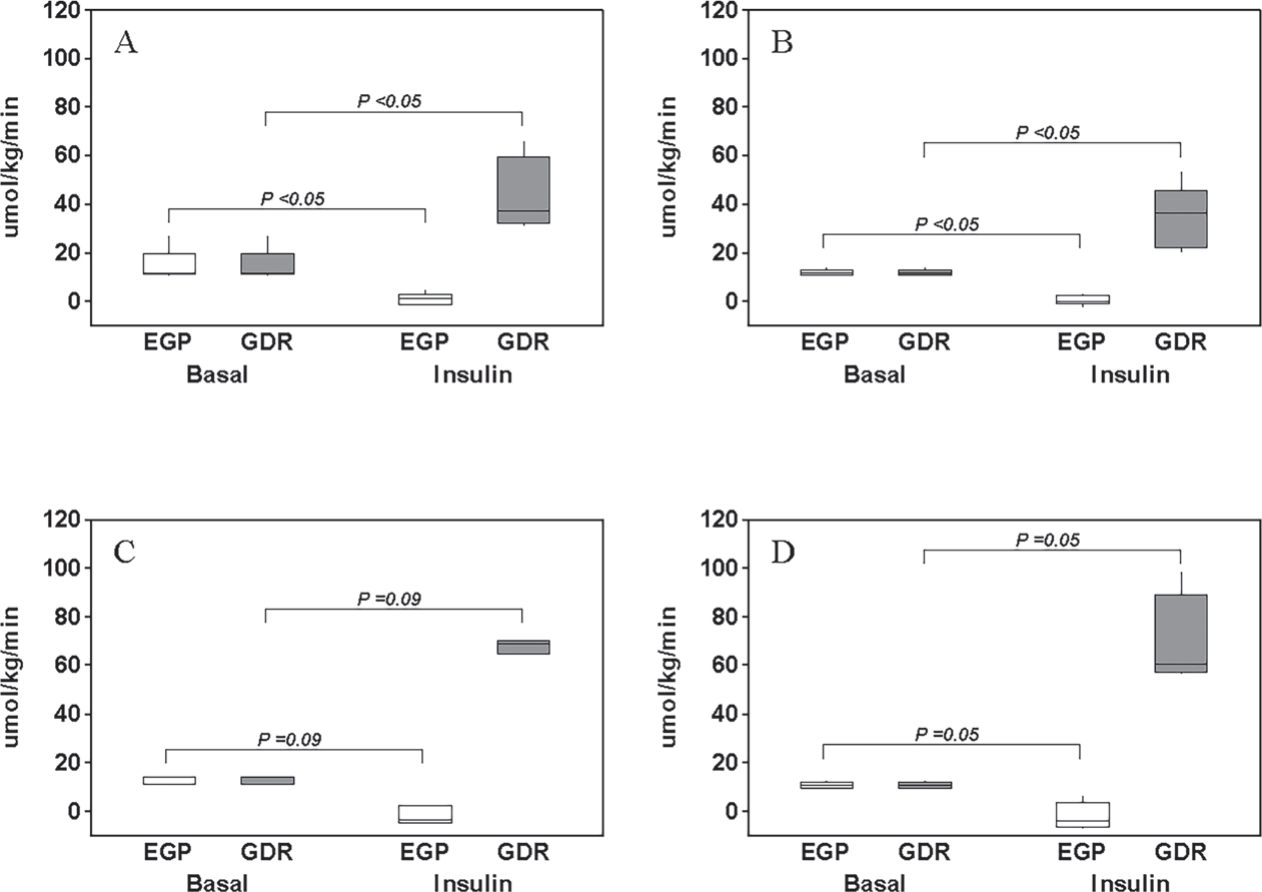
Plasma glucose turnover. Rates of endogenous glucose production (EGP) and whole-body glucose disposal (GDR) in the basal period (i.e., Basal) and following insulin infusion (i.e., Insulin). Insulin was infused at either 40 mU/m^2^/min in a control group (A) and a group with increased plasma branched-chain amino acid concentrations (B) or 80 mU/m^2^/min in a control group (C) and a group with increased plasma branched-chain amino acid concentrations (D). Boxes describe interquartile range (IQR; 1^st^ quartile—3^rd^ quartile) with the horizontal line in the box representing the median value. *P* values are for the comparison of the corresponding medians.

In the basal period, whole-body GDR and EGP were not different between the two groups in either the 40U or 80U experiments (*P* > 0.05). Further, there were no differences in hepatic insulin sensitivity (i.e., product of EGP and insulin concentration) in the basal period between the Control (62.9 (25.0–124.9)) and BCAA (53.6 (34.7–85.4)) groups when data from both the 40U and 80U experiments were pooled together (*P >* 0.05). During insulin infusion whole-body GDR increased in both the Control and BCAA groups, and in both the 40U and 80U experiments and as depicted in Fig. 2. At the same time, EGP decreased in the Control group during insulin infusion and the same response was also observed in the presence of increased plasma BCAA concentrations, and in both the 40U and 80U experiments (Fig. 2). The differences in GDR and EGP resulting from the insulin infusion in the Control group in the 80U experiment did not reach the stated statistical significance likely due to the limited number of subjects studied (N = 3; Fig. 2C).

**Fig 3:**
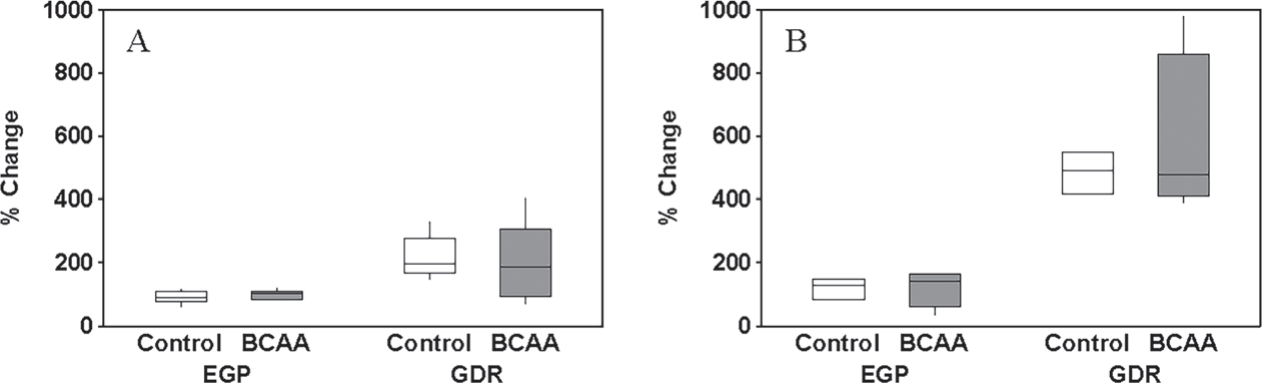
Insulin-stimulated changes in plasma glucose turnover. Changes from the basal period in the rates of endogenous glucose production (EGP) and whole-body glucose disposal (GDR) as a result of the insulin infusion at either 40 mU/m^2^/min (A) or 80 mU/m^2^/min (B) in the control group (Control) and the group with the increased plasma branched-chain amino acid (BCAA) concentrations. Boxes describe interquartile range (IQR; 1^st^ quartile—3^rd^ quartile) with the horizontal line in the box representing the median value.

Our main end-points were the changes in insulin-mediated whole-body GDR and EGP. Fig. 3 depicts these changes in the Control and BCAA groups, and in the 40U (Fig. 3A) and 80U (Fig. 3B) experiments. These changes in whole-body GDR or EGP were not different between the Control and BCAA group in either the 40U or 80U experiment (*P >* 0.05). These percent changes between the Control and BCAA groups were also not different in either of the two experiments (*P >* 0.05) when the GDR and EGP were adjusted to the prevailing plasma insulin concentrations.

### Glucose metabolic clearance

In the basal period, the glucose metabolic clearance was not different between the two groups in either the 40U or 80U experiments (*P* > 0.05). During insulin infusion in the 40U experiment, the rate of glucose metabolic clearance (ml/kg/min) increased in the Control from its basal value of 2.9 ± 0.5, 2.5 (2.2–3.8) to 8.8 ± 0.5, 8.6 (8.0–9.6) (*P <* 0.05), and in the BCAA group from its basal value of 2.5 ± 0.1, 2.4 (2.3–2.7) to 7.2 ± 1.2, 7.1 (4.7–9.8) (*P <* 0.05), with no difference in the insulin-mediated glucose metabolic clearance between groups (*P* > 0.05). Also, in the 40U experiment, the increase (%) in glucose metabolic clearance due to the insulin infusion in the BCAA group (192.3 ± 57.0, 152.0 (85.6–319.2)) was not different from that in the Control (228.7 ± 42.4, 254.0 (147.6–297.2); *P >* 0.05). After adjusting for the prevailing plasma insulin concentrations, the difference in glucose metabolic clearance rate percent change between groups became even smaller when compared to that of the insulin-unadjusted values (data not shown).

During insulin infusion in the 80U experiment, the rate of glucose metabolic clearance (ml/kg/min) increased in the Control from its basal value of 2.6 ± 0.2, 2.4 (2.4–3.0) to 13.5 ± 1.1, 13.7 (11.5–15.2) (*P =* 0.09), and in the BCAA group from its basal value of 2.6 ± 0.2, 2.6 (2.2–2.9) to 15.4 ± 1.5, 14.6 (13.0–18.6) (*P* = 0.05), with no difference in the insulin-mediated glucose metabolic clearance between groups (*P* > 0.05). Also, in the 80U experiment, the increase (%) in glucose metabolic clearance due to the insulin infusion in the BCAA group (506.7 ± 86.3, 481.5 (354.7–684.0)) was not different from that in the Control (416.0 ± 26.4, 405.1 (376.7–466.2); *P >* 0.05). After adjusting for the prevailing plasma insulin concentrations, the difference in glucose metabolic clearance rate percent change between groups became even smaller when compared to that of the insulin-unadjusted values (data not shown).

## Discussion

The role of increased plasma BCAA in modifying insulin sensitivity has been a topic of heavy debate. This is the first study to directly evaluate the effects of short-term exposure to increased BCAA levels on plasma glucose turnover in humans. The primary end-point of the study was the change in whole-body glucose disposal, reflecting primarily insulin sensitivity of glucose metabolism in muscle. Our results show that short-term exposure to increased plasma BCAA concentrations does not modify the insulin sensitivity of glucose metabolism in healthy, young humans.

By design, the BCAA infusion resulted in more than 2.5-fold increase in the concentration of plasma BCAA for a period of 6 hours, and at levels above those observed in either obesity [1,4] or the fed state associated with the ingestion of high protein meal [22]. We found that increasing the plasma insulin concentrations completely suppressed the EGP both in the control and BCAA groups. These findings agree with previous findings describing the response of EGP to insulin in association with the infusion of a mixture of amino acids that increased the plasma concentration of total amino acids (i.e., BCAA as well as the concentrations of essential and non-essential amino acids) [10,11,23]. It is recognized, however, that the present study, as well as the previous studies, may be limited in their ability to distinguish differences in EGP in the presence of increased plasma amino acids when plasma insulin concentrations are raised at levels that result in complete suppression of EGP. The product of EGP and plasma insulin concentration, which was used as an additional measure in evaluating hepatic insulin sensitivity [21] in the basal period and at a time when plasma insulin levels were relatively low in both the control and BCAA groups, indicated no differences in hepatic insulin sensitivity between the two groups. Therefore, the overall findings of the present studies suggest that short-term exposure to increased plasma BCAA concentrations does not impair insulin action in the liver in healthy young adults.

Although not a consistent finding [24], previous reports [10,11] have documented decreased insulin-stimulated whole-body GDR in association with an increase in the plasma concentration of total amino acids. Given that the increase in the plasma BCAA concentration in the present studies was comparable to that achieved in the previous investigations, the lack of a specific effect of increased plasma BCAA on decreasing whole-body GDR during insulin infusion in the present studies indicates that increased concentrations of other plasma amino acids, rather than BCAA alone, have greater role in impairing whole-body GDR. According to relevant evidence, decreased plasma glucose clearance in diabetic animals (i.e., pigs) during insulin stimulation is observed in parallel with increased clearance of almost all of the non-essential amino acids (NEAA), but not any of the BCAA [25]. This suggests that, among the plasma amino acids, NEAA metabolism may have greater role than the BCAA metabolism in inhibiting glucose disposal in skeletal muscle. *In vitro* work with muscle cells suggests that among the BCAA, although leucine had an inhibitory effect on insulin-mediated glucose uptake, the same effect was not observed with isoleucine or valine [26]. However, other essential amino acids (EAA) (i.e., methionine, threonine) and NEAA (i.e., cysteine, tyrosine) were potent inhibitors of the insulin-mediated muscle glucose uptake [26]. Recent studies in humans have shown that increased plasma concentrations of EAA alone decrease insulin sensitivity *in vivo* [27], suggesting a primarily role of particular amino acids within the EAA in decreasing insulin-mediated glucose disposal. However, Smith et al [28] have also recently shown in humans that, although increase in plasma concentrations of total amino acids following whey protein ingestion decreases insulin sensitivity, increase in leucine levels alone does not decrease insulin sensitivity. The present studies are in line with the latter finding and extent that evidence to all three BCAA, and by showing that increase in plasma total BCAA concentrations in young healthy subjects does not impair insulin sensitivity in muscle.

During the traditional hyperinsulinemic-euglycemic clamp, the insulin-mediated decrease in plasma total amino acid concentrations, including BCAA, is associated with greater peripheral glucose disposal than that seen when the plasma amino acid levels are maintained at their basal levels [14,29]. This creates a circumstance during which the effects of increased plasma amino acids on glucose turnover are compared to a rather non-physiological circumstance where the plasma amino acid concentrations are considerably below their basal levels. In the present study, the plasma total BCAA concentration was maintained during the insulin infusion in the control group in a way that it was not different from that measured in the basal/postabsortive levels. However, in all of the previous reports the plasma BCAA concentrations decreased more than 50% relative to that at the basal/postabsorptive conditions during the insulin infusion creating a circumstance of a non-physiological “control” [10,11,23]. Therefore, the difference in whole-body GDR between amino acid infusion and control in these previous reports [10,11,23] may relate in part to the greater rate of glucose disposal in the “control” experiments. By maintaining the overall plasma BCAA concentrations at the postabsorptive levels during the insulin infusion in the present study, we were able to clearly describe the role of the increase, per se, of the plasma BCAA concentrations on plasma glucose turnover.

Given the lack of an effect of increased plasma BCAA on inducing insulin resistance in the present study, the negative correlation reported previously between plasma BCAA and insulin sensitivity in humans [7] may reflect impaired ability to metabolize BCAA in insulin-resistant individuals. We have previously shown that the abundance of enzymes involved in the oxidation of BCAA in muscle is reduced in insulin-resistant individuals [30], and this can impair the utilization of BCAA leading to increased muscle and circulating BCAA levels. In addition to muscle, decreased activities or expression of key enzymes involved in BCAA catabolism in obesity/insulin-resistance have been reported in the liver and adipose tissue in animal models [31,32] and in the visceral fat in humans [33]. Increased concentrations of BCAA in Type 2 Diabetes have also been directly linked to decreased clearance of these amino acids from plasma [34]. There is previously reviewed evidence indicating that in a metabolic environment where free fatty acid concentrations are increased, which is a general observation in obesity/insulin-resistance, branched-chain ketoacid dehydrogenase activity is decreased [35]. Such observations can explain the negative correlation between plasma BCAA and insulin sensitivity, and where increased concentrations of BCAA are secondary to decreased ability to metabolize BCAA. Under these circumstances, and given that increased BCAA, per se, do not induce insulin resistance, increased plasma BCAA levels may be a “metabolic marker” of the metabolic environment associated with obesity and insulin resistance rather than a cause of insulin resistance.

It is noted that, in parallel with arguments implicating plasma BCAA as negative regulator of glucose metabolism, there is evidence arguing about a positive role of BCAA in the regulation of glucose metabolism [36–42]. Increased plasma BCAA concentrations, secondary to disruption of genes implicated in BCAA catabolism in experimental animal models, results in significant improvements in insulin sensitivity [39]. Supplementation with BCAA also improved plasma glucose homeostasis under a variety of abnormal metabolic conditions [40]. In an acute setting, orally administered isoleucine in normal/healthy mice improved glucose tolerance in the absence of any changes in plasma insulin concentrations [40], suggesting improved insulin sensitivity. Similar findings were obtained in studies with rats [43]. In the later report, however, separate studies with oral administration of leucine or valine did not result in improvements in insulin sensitivity. These latter findings are in line with the lack of an overall effect of the BCAA on altering insulin sensitivity in the present study in humans.

The parallel design employed in association with the total number of subjects studied per experiment may be considered limitations of the present study. However, and in addition to studying subjects that were fairly similar in terms of physiology and metabolic parameters, we compared differences in the changes, rather than direct differences, in insulin-stimulated glucose turnover between groups. Furthermore, the calculated sample size required to describe statistically significant difference between the control and BCAA groups across all data collected for the main-end point (i.e., GDR in Fig. 3) is 316 subjects per group (power value, 0.8; alpha value, 0.05). This is not surprising given that the overall difference in GDR between groups in the present study is less than 10%, and considerably smaller than the decrease (i.e., 25%) in GDR resulting from the elevation in total amino acid concentrations [11]. Smaller change in insulin-stimulated GDR in the present study when compared to that in the previous report [11], despite comparable increase in the concentration of BCAA, points in the direction for a major role of amino acids other that the total plasma BCAA in impairing insulin-stimulated GDR.

We studied young (i.e., 18–25 years old) healthy subjects, thus our findings do not address any effects of age or related metabolic conditions, such as obesity or diabetes, on the measured metabolic responses. Older/middle-age adults with Type 2 Diabetes have increased plasma BCAA concentrations when compared to their age-matched healthy controls [7], whereas adolescents with Type 2 Diabetes have in fact lower plasma BCAA concentrations when compared to healthy controls [44]. Furthermore, although there is a strong negative correlation between BCAA levels and insulin sensitivity in middle-age adults [7], no such correlation has been observed in adolescents [44]. Therefore, the lack of an effect of the increased plasma BCAA concentrations in inducing insulin resistance in the present study may be related to the young population studied. It is possible that in the metabolic background associated with type 2 Diabetes [7], or increased dietary fat intake [4], increased plasma BCAA may have distinct effects on plasma glucose turnover.

Increasing the plasma BCAA concentrations is a well-characterized phenomenon enhancing muscle protein anabolism in humans [12,13]. Furthermore, acute increase in the plasma BCAA concentrations appears to enhance muscle mitochondria function [45]. Our findings suggest that BCAA can be safely administered to improve muscle metabolic responses in healthy individuals without impairing glucose metabolism. Therefore, the findings of the present study become of great physiological and clinical importance when the acute effects of BCAA are considered in the context of their overall effects on muscle metabolism in humans.

In conclusion, acute increase in plasma concentrations of BCAA in healthy young subjects does not cause insulin resistance to glucose metabolism. The results of the present study are limited to short-term elevation in plasma BCAA levels. The effects of chronic elevation in the plasma BCAA levels on plasma glucose turnover in humans remain to be evaluated.
